# Applicability of Ash Wastes for Reducing Trace Element Content in *Zea mays* L. Grown in Eco-Diesel Contaminated Soil

**DOI:** 10.3390/molecules27030897

**Published:** 2022-01-28

**Authors:** Mirosław Wyszkowski, Jadwiga Wyszkowska, Natalia Kordala, Agata Borowik

**Affiliations:** 1 Department of Agricultural and Environmental Chemistry, University of Warmia and Mazury in Olsztyn, Łódzki 4 Sq., 10-727 Olsztyn, Poland; natalia.kordala@uwm.edu.pl; 2 Department of Soil Science and Microbiology, University of Warmia and Mazury in Olsztyn, Łódzki 3 Sq., 10-727 Olsztyn, Poland; jadwiga.wyszkowska@uwm.edu.pl (J.W.); agata.borowik@uwm.edu.pl (A.B.)

**Keywords:** Eco-Diesel oil, trace elements, coal ash, sludge ash, *Zea mays* L.

## Abstract

Among the large group of xenobiotics released into the environment, petroleum derivatives are particularly dangerous, especially given continuing industrial development and the rising demand for fuel. As increasing amounts of fly ash and sewage sludge are released, it becomes necessary to explore new methods of reusing these types of waste as reclamation agents or nutrient sources. The present study examined how soil contamination with Eco-Diesel oil (0; 10; 20 cm^3^ kg^−1^ soil) affected the trace-element content in the aerial parts of maize. Coal and sludge ashes were used as reclamation agents. Our study revealed that diesel oil strongly affected the trace-element content in the aerial parts of maize. In the non-amended group, Eco-Diesel oil contamination led to higher accumulation of the trace elements in maize (with the exception of Pb and Ni), with Cu and Mn content increasing the most. The ashes incorporated into the soil performed inconsistently as a reclamation agent. Overall, the amendment reduced Mn and Fe in the aerial parts of maize while increasing average Cd and Cu levels. No significant effect was noted for the other elements.

## 1. Introduction

Among the large group of xenobiotics released into the environment, petroleum derivatives are particularly dangerous, especially given continuing industrial development and the rising demand for fuel [1,2]. Diesel oil is a highly problematic pollutant, as it contains multiple toxic carcinogens and teratogens, which differ in their chemical structure and biodegradability. Diesel oil also has a high potential for migration and uncontrolled spread in the environment [3,4]. Compared to other petroleum derivatives, diesel oil is slower to evaporate and decompose [2], posing a challenge for environmental engineering.

Soil and water pollution with diesel oil is a global environmental issue [5,6] that poses a serious hazard to various ecosystems and human health [7]. Petroleum derivatives reduce yields and negatively affect the physicochemical parameters of soil, e.g., by increasing acidity and decreasing sorption capacity [8,9]. Improvers added to petrochemical products, which offer anti-corrosive, demulsifying, anti-foam, or antioxidant properties, may modify the effects of petroleum derivatives on the quality and condition of soil [10]. A study by Borowik et al. [11] demonstrated that Eco-Diesel Ultra increased organic carbon in contaminated soils and reduced the pool of available P and Mg. Furthermore, the oil inhibited the activity of nearly all soil enzymes (excluding catalase) and disrupted microbial diversity in the soil. Similar results were obtained by Borowik et al. [12], who further noted that Eco-Diesel Ultra oil was a persistent contaminant of soil environments.

Petroleum derivatives released into the environment adversely affect plant growth and development in contaminated soils [13,14,15,16] and alter the content of macroelements and trace elements in plant biomass [8,17,18]. Apart from negatively affecting the physicochemical [19,20], microbiological, and enzymatic properties of the soil [8,21,22], petroleum-derived hydrocarbons also pose a serious risk for human health due to their capacity to accumulate in plant and animal tissues [4].

Due to large-scale industrial activity, increasing urbanization, and the push towards a circular economy, steps must be taken towards recycling products/waste and limiting the consumption of natural resources [23]. Fly ash, municipal sewage sludge, and ash from the incineration thereof are all examples of waste harmful to the environment. Fly ash and bottom ash are by-products of combusting coal and lignite as fuel in all types of power plants [24]. Fly ash is a fine mineral waste product generated by hard coal combustion in thermal power stations. Fly ash can be repurposed in a number of ways. For many years it has been used as a value-added mineral admixture in cement, composite binders, and concrete [25], as well as in road construction, mine remediation, manufacture of mineral wool, metal recovery, and as a replacer for aggregates [24,26]. However, huge amounts of ash are still being stored/deposited and contribute to environmental pollution through foul air emissions, smog-forming dust, and leachate, through which trace elements are released into groundwater [27]. Furthermore, storing ash is costly and takes up space that could be put to other use. It is, therefore, necessary to explore new avenues of fly ash recycling [28]. One of the more effective ways to utilize coal fly ash is to convert it into zeolites (synthetic sorbents) [29]. It can also be used in agriculture [30,31] and for remediation of contaminated sites [28,32,33]. CaO-rich fly ash generated by combusting oil can be used as a liming agent in chemical precipitation [34]. Thanks to their aluminum silicate structure, fly ash particles can adsorb trace elements through ion exchange in aqueous solutions, thus reducing trace element availability [31]. Amending loamy soils with fly ash can produce persistent changes in soil composition, increase microporosity, and improve water retention [26,35]. The pH of fly ash varies from 4.5 to 12, depending on the sulfur content in the coal [31,36], making it a viable agent for increasing soil acidity or alkalinity, as needed [37,38]. The elemental content of fly ash makes it suitable as a source of macroelements (P, Ca, Mg) and essential trace elements (Cu, Fe, Mn, Mo, Zn, B) for plants, especially in soils with a limited supply [38,39], to boost plant growth and yields [30,40,41].

In turn, ash from municipal sludge incineration is a promising and sustainable recycled raw material for manufacturing phosphatic fertilizers [42,43]. The phosphorus content in ash dry mass ranges from 5% to 20% [44], being comparable to raw materials from medium-rich phosphorus deposits. Depending on the chemical composition of the municipal sludge and the particulars of the incineration process, the resultant ash can vary in terms of macroelement content (Ca, Mg, K) and trace element content (Fe, Si, Zn, Cu), including essential trace elements: Cd, Pb, As, Ni, and Cr [45]. As a fine-fraction waste material, sludge ash is mostly utilized in the production of asphalt pavement mixtures, cement mortar, brickmaking [46], as well as in agriculture and green land reclamation [47]. Further heat treatment or thermochemical treatment of sludge ash can remove organic and inorganic contaminants, including trace elements [44,48,49], opening up avenues to eco-friendly utilization.

Due to fly ash unique structure and content of nearly every nutrient necessary for plant metabolism (except for humus and nitrogen), it has been recognized as a potential tool in agriculture [30,50]. For the most part, fly ash is used as a substitute for mineral fertilizers to improve the physical, chemical, and biological properties of soils [31,51,52]. It is also used as an ameliorant and yield enhancer [36,53,54] and a potential means of reducing trace element accumulation in plants [55].

The aim of the present study was to assess the applicability of coal ash waste and sludge ash waste in mitigating the detrimental effect of Eco-Diesel oil on the trace-element content of *Zea mays* L.

## 2. Results

The contamination of the soil with Eco-Diesel oil, as well as soil amendments with reclamation substances, had a significant impact on the trace element content in the aerial parts of maize (Table 1, Table 2 and Table 3).

In the non-amended group, the aerial parts responded to Eco-Diesel contamination by accumulating more trace elements (with the exception of lead and nickel). At a dose of 20 cm^3^ kg^−1^ DM soil, copper and manganese were affected the most, with content found in the aerial parts of maize being almost 3.8 times and over 3.6 times higher than in the control (the non-contaminated group), respectively (Table 2 and Table 3). High doses of diesel oil significantly increased cadmium, chromium, zinc, iron, and cobalt content in the aerial parts of maize compared to the control. Pb and Ni content in the aerial parts of maize were inversely correlated with the oil dose. In the non-amended series, the content of these elements was lower by 22% and 25%, respectively, against the control (Table 1 and Table 2).

The effect of the reclamation agents (coal ash and sludge ash) on the chemical composition of maize was inconsistent (Table 1, Table 2 and Table 3). Application of coal ash at a dose of 2 g kg^−1^ DM soil increased the average cadmium content (by 34%) and reduced the content of manganese (by 7%) and iron (by 19%) in the aerial biomass, compared with the control (no-ash group). No significant effect was found for Cr, Zn, Cu, and Co. In turn, Pb and Ni content were affected but with no clear pattern.

Sludge ash was found to have a significant effect on Cr, Mn, Fe, and Co content, reducing their accumulation in the aerial biomass of maize (Table 1, Table 2 and Table 3). The reduction was the most pronounced for Fe—28% compared with the no-sludge series. The sludge ash, used as a reclamation agent, was found to have no effect on the Pb, Ni, and Zn. Amendment with ash at doses of 0.5 g kg^−1^ DM soil or less promoted copper accumulation. Similarly, cadmium content was elevated for amendments of up to 1 g kg^−1^ DM soil. At their highest doses (2 g kg^−1^ DM soil), cadmium content increased by 20%, and that of copper increased by 75% compared to the control (without amendments).

**Figure 1:**
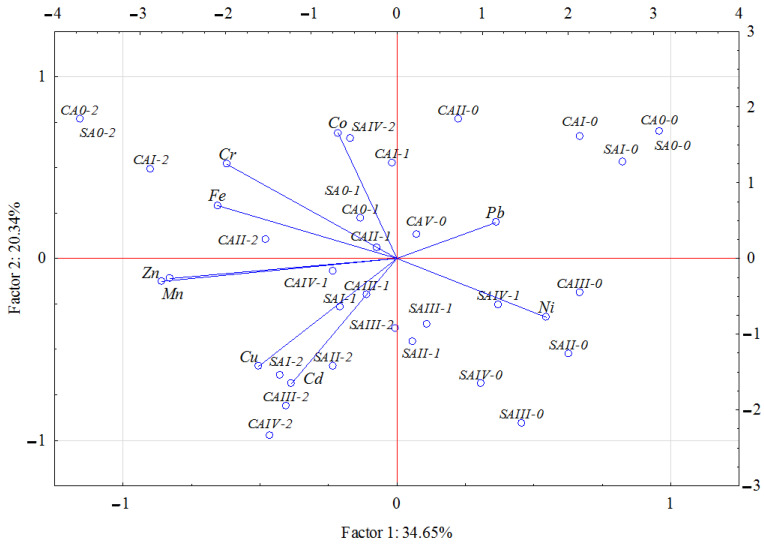
Content of trace elements in the above-ground parts of maize (*Zea mays* L.) illustrated with the PCA method. Key: vectors represent analyzed variables (content of Cd, Pb, Cr, Ni, Zn, Cu, Mn, Fe and Co); points show the samples with elements (*CA0*—without coal ash, *CAI*—0.25 g, *CAII*—0.5 g, *CAIII*—1.0 g, *CAIV*—2.0 g; ash per kg DM of soil; *SA0*—without sludge ash, *SAI*—0.25 g, *SAII*—0.5 g, *SAIII*—1.0 g, *SAIV*—2.0 g ash per kg of soil; *0*—0 g, *1*—10 g, *2*—20 g Eco-Diesel oil per kg DM of soil).

The correlation coefficients and the PCA results (Table 4 and Figure 1) confirmed significant correlations between the content of trace elements in the aerial biomass of maize. The strongest positive correlations were noted between Zn and Mn, between Cu and Cd, between Cu and Zn, and between Cr and Fe. In contrast, the strongest negative correlations were observed between Co and Cu and between Fe and Pb. Certain weaker negative correlations were also detected, namely between Mn and Ni and between Pb and Cd. The cumulative impact of Eco-Diesel oil and ash in soil on the content of selected trace elements in the aerial parts of maize is shown in Figure 1. The PCA showed that the principal components accounted for 54.99% of input data variation, producing two groups (group 1: chromium, nickel, zinc, copper, and manganese; group 2: cadmium, lead, and cobalt). The Pb and Ni vectors proved to be shorter than the others, indicating that these two components contributed least to the data set correlation. The dispersion of points in Figure 1 show that the reclamation agents generally had a positive (reducing) impact on trace-element content in the aerial parts of maize.

**Figure 2:**
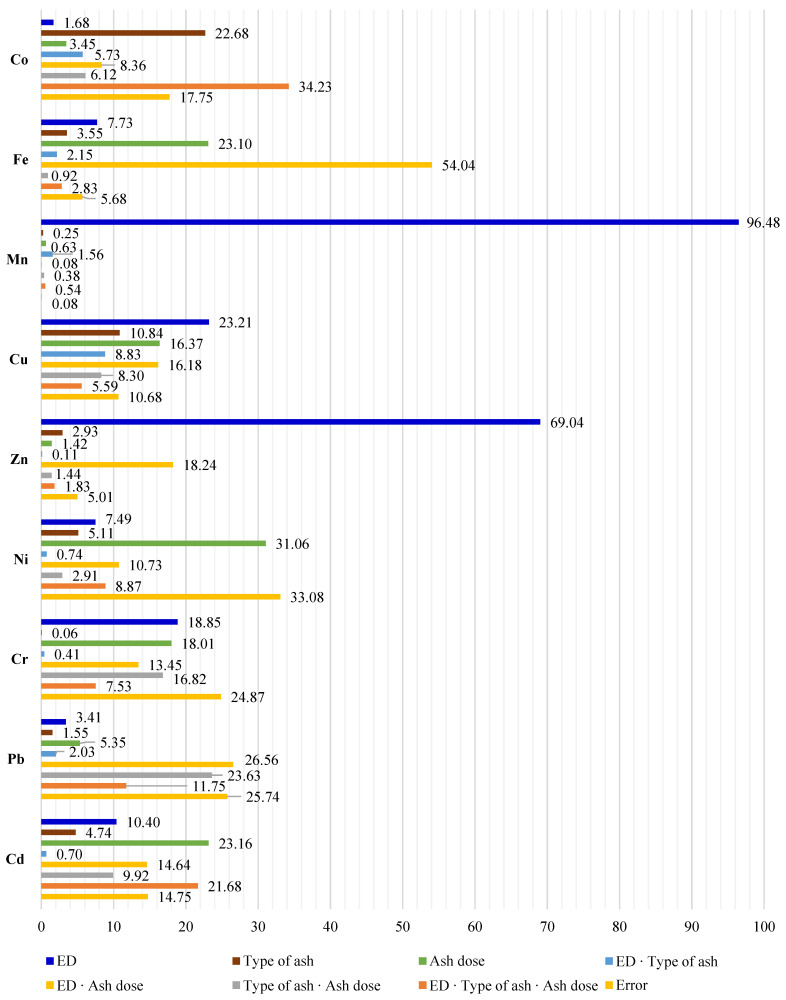
Percent contribution of tested factors according to the content of trace elements in above-ground parts of maize (*Zea mays* L.): ED—Eco-Diesel oil dose.

The observed variance ratio was determined as η2 using ANOVA. The trace-element content in maize proved to be most strongly determined by the diesel oil dose. The respective variable shares were 96.48%, 69.04%, 23.21%, and 18.85% for manganese, zinc, copper, and chromium (Figure 2). The contributions were much lower for the other elements, ranging between 1.68% (Co) and 10.40% (Cd). The effect of ash on the chemical composition of aerial parts of maize can be seen with element content ranging from 0.63% (Mn) to 31.06% (Ni) in the ash-amended group.

## 3. Discussion

Our study detected a positive correlation between soil contamination by Eco-Diesel and trace element content in the aerial biomass of maize (with the exception of lead and nickel). Wyszkowski and Sivitskaya [56] obtained similar results, and noted that increasing doses of diesel oil (up to 10 g kg^−1^ DM soil) resulted in elevated Cd, Mn, Cr, and Pb content in the aerial parts of maize. Rusin et al. [9] found that the application of diesel oil at 6 g kg^−1^ DM soil resulted in the accumulation of Fe, Mn, and Cd in aerial parts of wheat (*Triticum aestivum* L.). In another experiment, the same research group [57] concluded that petroleum caused an increase in Pb and Mn content in the leaves of broad bean plants, which partly matches the results of the present study. Petroleum derivatives modify the content of trace elements in the soil, thus indirectly affecting their content in plant organs [58]. The uptake of available trace elements by plants tends to correlate positively with their content in the soil. Petroleum products have been shown to increase Cd, Pb, Cu, and Mn content in the soil [59,60] and reduce soil pH [16,61,62,63]. This, in turn, may increase the phytoavailability of trace elements, their uptake biodynamics, and their translocation to aerial plant organs [64,65].

Incorporating coal ash into the soil led to reduced Mn and Fe accumulation in all experimental variants. An inverse effect was observed for cadmium, with the aerial parts of maize containing significantly elevated Cd. No significant effect was noted for the other assayed elements. These findings are corroborated by Karmakar et al. [66], who produced elevated selenium and cadmium content in rice grain and straw by adding 5 and 10 Mg ha^−1^ of fly ash. Tsadilas et al. [67,68] showed fly ash to have a similar effect on Mn and Fe in the aerial parts of maize. Dash and Sahoo [69] studied the effects of fly ash amendments (at 20, 40, 80, and 100%) on the growth and chemical composition of the edible parts of leaf mustard (*Brassica juncea*). This research group noted improved growth and yields, combined with increased accumulation of trace elements (Cu, Cd, Cr and Pb) against the control, though still within WHO recommended limits. Gond et al. [70] demonstrated that amending the soil with 180 Mg ha^−1^ of fly ash improved the soil’s physical properties and boosted the growth and yields of *Solanum melongena*. Gupta and Sinha [71] found that mung bean grown in the soil blended with fly ash (at 10 and 25%) exhibited increased Fe, Co, and Cd translocation. Furthermore, they found that the sprouts and beans contained Pb and Ni content that was several times higher than in the control plants. Though the amendment of soil with fly ash increases trace element content (especially in heavy soils), this effect only extends to their non-available forms [68], most likely due to the soil-liming effect of the amendment. This may explain why the present study showed no change in the aerial biomass of maize after amendment with fuel ash.

By contrast, sludge ash addition to the soil significantly decreased manganese, iron, chromium, and cobalt content in the aerial biomass while increasing cadmium and copper content. Other researchers have also observed this pattern, finding that tissues of plants grown on sludge ash-treated soil accumulate more cadmium, lead [72], molybdenum, boron, and copper [73]. Similarly, Sormunen et al. [74] indicated significantly increased content of copper and zinc in ryegrass and barley grown in soil blended with sludge ash, though these concentrations did not exceed content potentially toxic to the plants. The mobility of soil trace elements is partly determined by chloride content. Chlorides are prevalent in ashes (0.04–0.099%) [35,75] and can form complexes with some trace elements, increasing the mobility and phytoavailability of the latter [76]. Pyrolysis (>500 °C) of sludge produces biochar [77], which may be used as a promising alternative method of soil remediation. Waqas et al. [78] noted a significant reduction in the availability of PAHs (polycyclic aromatic hydrocarbons) and the content of trace elements upon application of biochar at a 10% ratio. The treatment also successfully reduced trace element accumulation (with the exception of cadmium and zinc) in cucumber biomass.

Both sludge and fuel ash can potentially be used as fertilizers and liming materials [79]. According to the Intergovernmental Panel on Climate Change (IPCC), the entirety of carbon contained in agrilime is eventually released into the atmosphere as CO_2_, exacerbating global warming. Therefore, replacing agrilime with fly ash may reduce net CO_2_ emission and thus counteract climate change [34]. Multiple experiments [80,81] provide further support for the effectiveness of sludge-fly ash mixtures as a tool for soil conditioning and increasing trace element bioavailability. The agricultural applicability of both fly ash and municipal waste ash is determined by the soil amendment ratio, type of crop, and the soil environment [76]. The main barrier to ash recycling lies in the stringent standards in force, which regulate the physical properties and chemical composition of recycled ash—in particular, the content of trace elements and radioactive substances capable of accumulating in the food chain [82]. Therefore, more research on the subject is needed.

Results of research also indicate a limiting impact of many other materials in limiting the content of trace elements in plants, e.g., chitosan/silver/Mn-Mg ferrite nanocomposite [83], indigenous garlic peel and mercerized garlic peel [84], etc.

## 4. Materials and Methods

### 4.1. Experimental Design

The study consisted of a pot vegetation experiment conducted in a greenhouse facility of the University of Warmia and Mazury in Olsztyn (north-eastern Poland). The experiment was conducted in a soil formation classified as loamy sand according to the International Union of Soil Sciences and the United States Department of Agriculture [85] (particle size distribution: >0.05 mm sand—51.20%, 0.002–0.05 mm silt—45.34% and <0.002 mm clay—1.46%). The soil had the following properties: pH in 1 M KCl L^−1^—6.73; hydrolytic acidity of 8.00 mM(+) kg^−1^; total exchangeable bases—126.7 mM(+) kg^−1^; cation exchange capacity of 134.7 mM(+) kg^−1^; base saturation of 94.6%; total organic carbon of 12.6 g kg^−1^; total nitrogen of 1.08 g kg^−1^; available phosphorus forms of 83.28 mg P kg^−1^; potassium of 178.50 mg K kg^−1^, and magnesium of 3.20 mg Mg kg^−1^ DM of soil. The experiment was designed to determine the impact of soil pollution with Eco-Diesel oil (in doses of 0, 10, and 20 cm^3^ 1 kg^−1^ DM soil) on the trace-element content in the aerial parts of maize. To mitigate any negative impact of the oil on plants, the soil was amended with coal ash waste or sludge ash waste in doses of: 0; 0.25; 0.5; 1; and 2 g 1 kg^−1^ soil. The following macronutrients were added to each pot: nitrogen—112 mg [CO(NH_2_)_2_], phosphorus—39 mg [KH_2_PO_4_], potassium—112 mg [KH_2_PO_4_ + KCl], and magnesium—15 mg 1 kg^−1^ DM soil [MgSO_4_·7H_2_O].

The trace elements content in coal/sludge ash used in the experiment are presented in Table 5. The soil was blended with Eco-Diesel oil, the ashes and mineral fertilizer (soil weight: 2.8 kg), then put into polyethylene pots with dimensions of 15.5 cm (height), 14 cm (lower diameter of the pot), 17 cm (upper diameter of the pot). Afterwards, maize (*Zea mays* L., LG 32.58) was sown at six plants per pot. The experiment was conducted in four repetitions, with stable moisture content maintained during plant growth (at 60% of the capillary water capacity). The maize was harvested and sampled for analysis after panicle emergence (BBCH 59).

### 4.2. Methodology

Samples of the aerial parts of maize were cut up, dried at a temperature of 60 °C in Binder FED720 drying and heating chamber (Binder GmbH, Tuttlingen, Germany), and ground using the cutting mill SM 200 (Retsch GmbH, Haan, Germany). They (0.3 g) were then wet-digested in 65% nitric acid (HNO_3_ of analytical grade, 1.40 g cm^−3^ density) in Xpress Teflon^®^ vessels placed into a MARS 6 microwave system by CEM Corporation, Matthews, NC, USA, according to the protocol US-EPA3051 [86]. The trace element composition (Cd, Pb, Cr, Ni, Zn, Cu, Mn, Fe, and Co) was assayed using a SpectrAA 240FS spectrophotometer(Varian Inc., Mulgrave, VIC, Australia) via atomic absorption spectrometry [87]. For quality control of the determination of the trace elements content of samples, standard solutions of the Fluka company (Cd 51994, Pb 16595, Cr 02733, Ni 42242, Zn 188227, Cu 38996, Mn 63534, Fe 16596, Co 119785.0100) were used, and certified reference materials (NCS ZC 73030 from Chinese National Analysis Centre for Iron and Steel, Beijing, China) were also analyzed.

The following soil parameters were analyzed prior to setting up the experiment: particle size distribution was determined using the areometric (sieve-sedimentation) method [88], pH in 1 M KCl—using the potentiometric method with a pH 538 WTW meter and a pH SenTix61 electrode—WTW, Wroclaw, Poland (10 g soil/1 M KCL solution ratio—1:2.5) [89], hydrolytic acidity—HAC—using Kappen’s method (40 g of soil, extraction in 0.5 M calcium acetate, tritration with 0.1 M NaOH) [90], total exchangeable bases—TEB—using Kappen’s method (20 g of soil, extraction in 0.1 M hydrochloric acid, tritration with 0.1 M NaOH) [90], total nitrogen—using the Kjeldahl method (10 g of soil, mineralization in concentrated 96% sulfuric acid with the addition of 30% H_2_O_2_ using Speed-Digester K-439—BÜCHI Labortechnik AG, Flawil, Switzerland, destilation in KjelFlex K-360—BÜCHI Labortechnik AG, Flawil, Switzerland, tritration with 0.1 M NaOH) [91], total organic carbon—using Turin’s method (0.2 g of soil, extraction in 0.068 M K_2_Cr_2_O_7_ and H_2_SO_4_ mixture with the addition of Ag_2_SO_4_ and tritration with 0.1 M Mohr’s salt) [92], available phosphorus using the Egner–Riehm method (5 g of soil, extraction in 0.04 M calcium lactate and addition of 5% (NH_4_)_6_Mo_7_O_24_·4H_2_O and SnCl_2_, assayed using Shimadzu UV-1900 spectrophotometer—Shimadzu Corporation, Kyoto, Japan) [93], available potassium—using the Egner–Riehm method (5 g of soil, extraction in 0.04 M calcium lactate and addition of 10% oxalic acid—(COOH)_2_, assayed using a SpectrAA 240FS spectrophotometer—Varian Inc., Mulgrave, VIC, Australia) [93], and available magnesium—using the Schachtschabel method (5 g of soil, extraction in 0.0125 M calcium chloride, assayed using a SpectrAA 240FS spectrophotometer—Varian Inc., Mulgrave, VIC, Australia) [94]. The HAC and TEB values served to calculate cation exchange capacity (CEC) and base saturation (BS) with the following formulas: CEC = TEB + HAC and BS = (TEB/CEC) × 100 [88].

### 4.3. Statistical Analysis

The statistical treatment of results was conducted using the Statistica [95] package (TIBCO Software Inc., Palo Alto, CA, USA) and encompassed: two-way analysis of variance (ANOVA), Pearson linear correlation coefficients, principal component analysis (PCA), and the observed variance ratio with the coefficient η2 (using ANOVA).

## 5. Conclusions

Soil contamination with Eco-Diesel oil altered the chemical composition of maize. In the series without the reclamation agents, Eco-Diesel oil contamination increased trace elements (with the exception of Pb and Ni) in the aerial parts of maize. The effect of Eco-Diesel oil was most pronounced for manganese and copper.

The ashes—incorporated into the soil to mitigate the negative impact of petroleum derivatives—were successful in reducing most of the trace elements in maize, with the exception of Cd and Cu. Sludge ash was more effective in boosting tolerance to diesel oil eco-toxicity in maize than coal ash. Sludge ash reduced the content of four of the trace elements (Mn, Fe, Cr, and Co) in the maize biomass (vs. the control). By contrast, coal ash had the same effect only for two of the elements (Mn, Fe) in the maize biomass.

Ashes, especially sewage sludge ash, can be an effective reclamation agents of soils contaminated with Eco-Diesel oil.

## Figures and Tables

**Table 1:** Content of cadmium (Cd), lead (Pb), and chromium (Cr) in above-ground parts of maize—*Zea mays* L. (mg kg^−1^ DM).

Eco-Diesel Oil Dose (cm^3^ kg^−1^ DM of Soil)	Ash Dose (g kg^−1^ DM of Soil)					Average
	0	0.25	0.5	1	2	
Cadmium (Cd)						
Coal ash						
0	0.158 *^a^*	0.182 *^ab^*	0.187 *^ab^*	0.207 *^ab^*	0.226 *^ab^*	0.192 *^A^*
10	0.198 *^ab^*	0.177 *^ab^*	0.233 *^ab^*	0.239 *^ab^*	0.232 *^ab^*	0.226 *^A^*
20	0.179 *^ab^*	0.230 *^ab^*	0.203 *^ab^*	0.237 *^ab^*	0.256 *^b^*	0.210 *^A^*
Average	0.178 *^I^*	0.196 *^I,II^*	0.208 *^I,II^*	0.228 *^II^*	0.238 *^II^*	0.210
Sludge ash						
0	0.158 *^ab^*	0.148 *^a^*	0.186 *^abc^*	0.220 *^bc^*	0.218 *^bc^*	0.186 *^A^*
10	0.198 *^abc^*	0.216 *^bc^*	0.187 *^abc^*	0.207 *^abc^*	0.188 *^abc^*	0.199 *^A^*
20	0.179 *^ab^*	0.217 *^bc^*	0.250 ^c^	0.214 *^abc^*	0.156 *^ab^*	0.203 *^A^*
Average	0.178 *^I^*	0.194 *^I,II^*	0.208 *^I,II^*	0.214 *^II^*	0.187 *^I,II^*	0.196
Lead (Pb)						
Coal ash						
0	0.462 *^a^*	0.487 *^a^*	0.385 *^a^*	0.345 *^a^*	0.366 *^a^*	0.409 *^A^*
10	0.473 *^a^*	0.483 *^a^*	0.480 *^a^*	0.406 *^a^*	0.363 *^a^*	0.441 *^A^*
20	0.362 *^a^*	0.423 *^a^*	0.459 *^a^*	0.431 *^a^*	0.348 *^a^*	0.405 *^A^*
Average	0.432 *^II,III^*	0.464 *^III^*	0.441 *^II,III^*	0.394 *^I^*^,*II*^	0.359 *^I^*	0.418
Sludge ash						
0	0.462 *^a^*	0.455 *^a^*	0.432 *^a^*	0.434 *^a^*	0.408 *^a^*	0.438 *^A^*
10	0.473 *^a^*	0.363 *^a^*	0.389 *^a^*	0.475 *^a^*	0.469 *^a^*	0.434 *^A^*
20	0.362 *^a^*	0.402 *^a^*	0.453 *^a^*	0.431 *^a^*	0.463 *^a^*	0.422 *^A^*
Average	0.432 *^I^*	0.407 *^I^*	0.425 *^I^*	0.447 *^I^*	0.447 *^I^*	0.431
Chromium (Cr)						
Coal ash						
0	1.062 *^a^*	1.164 *^a^*	1.329 *^a^*	0.813 *^a^*	0.888 *^a^*	1.051 *^A^*
10	1.192 *^a^*	1.360 *^a^*	1.239 *^a^*	0.967 *^a^*	1.068 *^a^*	1.165 *^A^*
20	1.760 *^a^*	1.822 *^a^*	1.267 *^a^*	1.003 *^a^*	0.770 *^a^*	1.324 *^A^*
Average	1.338 *^I^*	1.449 *^I^*	1.278 *^I^*	0.928 *^I^*	0.909 *^I^*	1.180
Sludge ash						
0	1.062 *^a^*	1.087 *^a^*	0.969 *^a^*	1.204 *^a^*	0.993 *^a^*	1.063 *^A^*
10	1.192 *^a^*	1.186 *^a^*	0.990 *^a^*	1.205 *^a^*	1.105 *^a^*	1.136 *^A^*
20	1.760 *^b^*	1.252 *^a^*	1.242 *^a^*	1.307 *^ab^*	1.363 *^ab^*	1.385 *^B^*
Average	1.338 *^II^*	1.175 *^I^*^,*II*^	1.067 *^I^*	1.239 *^I,II^*	1.154 *^I,II^*	1.194

Values denoted by the different letters and Roman numbers are significantly different at *p* ≤ 0.01: *^A^^–B^* for Eco-Diesel oil dose, *^I^^–III^* for ash dose, and *^a^^–c^* for the interaction between Eco-Diesel oil dose and ash dose (Anova, Tukey’s HSD test).

**Table 2:** Content of nickel (Ni) zinc (Zn), and copper (Cu) in above-ground parts of maize—*Zea mays* L. (mg kg^−1^ DM).

Eco-Diesel Oil Dose (cm^3^ kg^−1^ DM of Soil)	Ash Dose (g kg^−1^ DM of Soil)					Average
	0	0.25	0.5	1	2	
Nickel (Ni)						
Coal ash						
0	1.368 *^ab^*	1.253 *^ab^*	1.151 *^ab^*	1.983 *^b^*	0.818 *^a^*	1.315 *^A^*
10	1.338 *^ab^*	1.29 *^ab^*	1.160 *^ab^*	1.396 *^ab^*	1.068 *^ab^*	1.251 *^A^*
20	1.024 *^ab^*	1.155 *^ab^*	1.054 *^ab^*	1.331 *^ab^*	1.262 *^ab^*	1.165 *^A^*
Average	1.243 *^I,II^*	1.234 *^I,II^*	1.122 *^I,II^*	1.570 *^II^*	1.049 *^I^*	1.244
Sludge ash						
0	1.368 *^a^*	1.530 *^a^*	1.608 *^a^*	1.710 *^a^*	1.146 *^a^*	1.472 *^A^*
10	1.338 *^a^*	1.229 *^a^*	1.421 *^a^*	1.747 *^a^*	1.463 *^a^*	1.440 *^A^*
20	1.024 *^a^*	1.093 *^a^*	1.197 *^a^*	1.680 *^a^*	1.160 *^a^*	1.231 *^A^*
Average	1.243 *^I^*	1.284 *^I^*	1.409 *^I^*	1.712 *^I^*	1.256 ^*I*^	1.381
Zinc (Zn)						
Coal ash						
0	10.78 *^a^*	12.87 *^ab^*	13.11 *^abc^*	15.24 *^bcd^*	16.11 *^bcde^*	13.62 *^A^*
10	21.72 *^g^*	20.34 *^fg^*	18.84 *^defg^*	16.74 *^cdef^*	17.10 *^def^*	18.95 *^B^*
20	20.18 *^fg^*	19.98 *^fg^*	21.17 *^g^*	19.67 *^efg^*	21.88 *^g^*	20.58 *^C^*
Average	17.56 *^I^*	17.73 *^I^*	17.71 *^I^*	17.22 *^I^*	18.36 *^I^*	17.72
Sludge ash						
0	10.78 *^a^*	10.19 *^a^*	11.22 *^a^*	12.91 *^ab^*	15.05 *^abc^*	12.03 *^A^*
10	21.72 *^c^*	19.07 *^bc^*	17.42 *^abc^*	16.90 *^abc^*	14.49 *^abc^*	17.92 *^B^*
20	20.18 *^c^*	20.50 *^c^*	16.55 *^abc^*	19.39 *^bc^*	20.38 *^c^*	19.40 *^B^*
Average	17.56 *^I^*	16.59 *^I^*	15.06 *^I^*	16.40 *^I^*	16.64 *^I^*	16.45
Copper (Cu)						
Coal ash						
0	0.828 *^a^*	1.241 *^ab^*	1.572 *^abc^*	1.655 *^abc^*	2.069 *^abcd^*	1.473 *^A^*
10	1.903 *^abc^*	2.566 *^bcd^*	2.152 *^abcd^*	2.648 *^bcd^*	2.566 *^bcd^*	2.367 *^B^*
20	3.145 *^cd^*	2.897 *^cd^*	2.483 *^bcd^*	3.559 *^d^*	2.731 *^bcd^*	2.963 *^C^*
Average	1.959 *^I^*	2.235 *^I^*	2.069 *^I^*	2.621 *^I^*	2.455 *^I^*	2.268
Sludge ash						
0	0.828 *^a^*	2.400 *^ab^*	3.559 *^b^*	3.562 *^b^*	2.731 *^ab^*	2.616 *^A^*
10	1.903 *^ab^*	2.559 *^ab^*	3.393 *^b^*	3.062 *^b^*	2.897 *^b^*	2.763 *^A^*
20	3.145 *^b^*	3.062 *^b^*	3.310 *^b^*	2.814 *^b^*	2.566 *^ab^*	2.979 *^A^*
Average	1.959 *^I^*	2.674 *^I,II^*	3.421 *^II^*	3.146 *^II^*	2.731 *^I,II^*	2.786

Values denoted by the different letters and Roman numbers are significantly different at *p* ≤ 0.01: *^A^^–C^* for Eco-Diesel oil dose, *^I^^–II^* for ash dose, and *^a^^–g^* for the interaction between Eco-Diesel oil dose and ash dose (Anova, Tukey’s HSD test).

**Table 3:** Content of manganese (Mn), iron (Fe), and cobalt (Co) in above-ground parts of maize—*Zea mays* L. (mg kg^−1^ DM).

Eco-Diesel Oil Dose (cm^3^ kg^−1^ DM of Soil)	Ash Dose (g kg^−1^ DM of Soil)					Average
	0	0.25	0.5	1	2	
Manganese (Mn)						
Coal ash						
0	45.22 *^b^*	34.38 *^a^*	39.23 *^ab^*	33.48 *^a^*	32.29 *^a^*	36.92 *^A^*
10	106.97 ^e^	99.06 *^cd^*	105.82 *^de^*	99.98 *^cde^*	96.55 *^c^*	101.68 *^B^*
20	162.74 *^f^*	159.19 *^f^*	173.79 *^h^*	170.55 *^gh^*	163.91 *^fg^*	166.04 *^C^*
Average	104.98 *^III^*	97.54 *^I^*	106.28 *^III^*	101.34 *^II^*	97.58 *^I^*	101.54
Sludge ash						
0	45.22 *^a^*	41.13 *^a^*	38.31 *^a^*	38.13 *^a^*	37.27 *^a^*	40.01 *^A^*
10	106.97 *^bc^*	112.98 *^c^*	109.53 *^bc^*	101.76 *^b^*	99.02 *^b^*	106.05 *^B^*
20	162.74 *^f^*	151.42 *^e^*	137.98 *^d^*	135.42 *^d^*	132.67 *^d^*	144.05 *^C^*
Average	104.98 *^III^*	101.84 *^III^*	95.27 *^II^*	91.77 *^I,II^*	89.65 *^I^*	96.70
Iron (Fe)						
Coal ash						
0	33.66 *^a^*	39.46 *^ab^*	47.57 *^ab^*	40.69 *^ab^*	41.99 *^ab^*	40.67 *^A^*
10	50.29 *^b^*	33.73 *^a^*	36.75 *^ab^*	49.53 *^b^*	42.93 *^ab^*	42.65 *^A^*
20	72.07 *^c^*	50.58 *^b^*	43.48 *^ab^*	42.43 *^ab^*	42.07 *^ab^*	50.13 *^B^*
Average	52.01 *^II^*	41.26 *^I^*	42.60 *^I^*	44.22 *^I^*	42.33 *^I^*	44.48
Sludge ash						
0	33.66 *^ab^*	35.00 *^ab^*	40.36 *^ab^*	40.75 *^ab^*	41.85 *^ab^*	38.32 *^A^*
10	50.29 *^b^*	37.39 *^ab^*	36.30 *^ab^*	48.87 *^b^*	34.96 *^ab^*	41.56 *^A^*
20	72.07 *^c^*	39.13 *^ab^*	35.40 *^ab^*	29.13 *^a^*	35.83 *^ab^*	42.31 *^A^*
Average	52.01 *^II^*	37.17 *^I^*	37.35 *^I^*	39.58 *^I^*	37.55 *^I^*	40.73
Cobalt (Co)						
Coal ash						
0	2.561 *^a^*	2.645 *^a^*	2.829 *^a^*	2.829 *^a^*	2.863 *^a^*	2.745 *^A^*
10	2.511 *^a^*	2.935 *^a^*	2.714 *^a^*	2.885 *^a^*	2.860 *^a^*	2.781 *^A^*
20	2.788 *^a^*	3.019 *^a^*	2.543 *^a^*	2.406 *^a^*	2.446 *^a^*	2.640 *^A^*
Average	2.620 *^I^*	2.866 *^I^*	2.695 *^I^*	2.707 *^I^*	2.723 *^I^*	2.722
Sludge ash						
0	2.561 *^bc^*	2.804 *^c^*	2.234 *^abc^*	1.911 *^ab^*	1.858 *^a^*	2.274 *^A^*
10	2.511 *^abc^*	2.518 *^abc^*	2.455 *^abc^*	2.340 *^abc^*	2.322 *^abc^*	2.429 *^AB^*
20	2.788 *^c^*	2.004 *^ab^*	2.459 *^abc^*	2.567 *^bc^*	2.882 *^c^*	2.540 *^B^*
Average	2.620 *^II^*	2.442 *^I,II^*	2.383 *^I,II^*	2.273 *^I^*	2.354 *^I,II^*	2.414

Values denoted by the different letters and Roman numbers are significantly different at *p* ≤ 0.01: *^A^^–C^* for Eco-Diesel oil dose, *^I^^–III^* for ash dose, and *^a^^–h^* for the interaction between Eco-Diesel oil dose and ash dose (Anova, Tukey’s HSD test).

**Table 4:** Correlation coefficients (r) between content of trace elements in above-ground parts of maize—*Zea mays* L.

Trace Elements	Pb	Cr	Ni	Zn	Cu	Mn	Fe	Co
Cd	−0.279 *	−0.185	−0.175	0.362 **	0.364 **	0.299 *	0.011	−0.182
Pb		0.063	0.203	−0.131	−0.127	−0.092	−0.319 *	−0.140
Cr			−0.225	0.312 *	0.177	0.412 **	0.495 **	0.244
Ni				−0.259 *	−0.015	−0.282 *	−0.202	−0.187
Zn					0.292 *	0.804 **	0.338 **	0.133
Cu						0.465 **	0.207	−0.319 *
Mn							0.332 *	0.091
Fe								0.159

Significant at ** *p* ≤ 0.01, * *p* ≤ 0.05.

**Table 5:** Trace element content in coal ash waste and sludge ash waste applied in the experiment (mg kg^−1^ DM).

Parameters	Unit	Coal Ash	Sludge Ash
Cadmium	mg kg^−1^ DM	2.972	4.984
Lead	mg kg^−1^ DM	94.47	190.49
Chromium	mg kg^−1^ DM	16.87	75.18
Nickel	mg kg^−1^ DM	328.20	95.44
Zinc	mg kg^−1^ DM	54.49	940.09
Copper	mg kg^−1^ DM	81.03	811.00
Manganese	mg kg^−1^ DM	519.51	417.97
Iron	g kg^−1^ DM	74.28	37.15
Cobalt	mg kg^−1^ DM	248.05	47.70

## Data Availability

All data are available in the manuscripts and from the authors.
